# Cortical Network Models of Firing Rates in the Resting and Active States Predict BOLD Responses

**DOI:** 10.1371/journal.pone.0144796

**Published:** 2015-12-14

**Authors:** Maxwell R. Bennett, Les Farnell, William G. Gibson, Jim Lagopoulos

**Affiliations:** 1 The Brain and Mind Research Institute, University of Sydney, Sydney, NSW, Australia; 2 The Centre for Mathematical Biology, University of Sydney, Sydney, NSW, Australia; 3 The School of Mathematics and Statistics, University of Sydney, Sydney, NSW, Australia; University of British Columbia, CANADA

## Abstract

Measurements of blood oxygenation level dependent (BOLD) signals have produced some surprising observations. One is that their amplitude is proportional to the entire activity in a region of interest and not just the fluctuations in this activity. Another is that during sleep and anesthesia the average BOLD correlations between regions of interest decline as the activity declines. Mechanistic explanations of these phenomena are described here using a cortical network model consisting of modules with excitatory and inhibitory neurons, taken as regions of cortical interest, each receiving excitatory inputs from outside the network, taken as subcortical driving inputs in addition to extrinsic (intermodular) connections, such as provided by associational fibers. The model shows that the standard deviation of the firing rate is proportional to the mean frequency of the firing when the extrinsic connections are decreased, so that the mean BOLD signal is proportional to both as is observed experimentally. The model also shows that if these extrinsic connections are decreased or the frequency of firing reaching the network from the subcortical driving inputs is decreased, or both decline, there is a decrease in the mean firing rate in the modules accompanied by decreases in the mean BOLD correlations between the modules, consistent with the observed changes during NREM sleep and under anesthesia. Finally, the model explains why a transient increase in the BOLD signal in a cortical area, due to a transient subcortical input, gives rises to responses throughout the cortex as observed, with these responses mediated by the extrinsic (intermodular) connections.

## Introduction

The cortical metabolic rate of glucose oxidation (CMR_glc(ox)_) gives a good measure of the average synaptic and firing activity in a region of cortex [1], although it is unclear that this can be used as a surrogate for the resting-state blood oxygenation level dependent (BOLD) signal measured with fMRI, as is often taken to be the case [2, 3]. This is because the fMRI signal provides a measure of the fluctuations about the average firing rate in a region of cortex, not the absolute size of the frequency [4]. Nevertheless, it has been shown that the average amplitude of the resting-state CMR_glc(ox)_ in a cortical area is proportional to the average resting-state BOLD there [3], in studies in which the global signal has not been regressed out. This observation implies that fluctuations in the firing rate in a region of interest are proportional to the average firing rate. We have found that this is so in our cortical model, thus providing the opportunity to determine how it might arise.

Correlations of BOLD signals between brain areas of subjects in the resting-state have been taken to characterize resting-state functional networks [5], with the correlations probably mediated in many cases by synapses formed by associational fiber axons [6, 7]. During sleep and anesthesia some of these correlations are lost, possibly due to changes in synaptic transmission mediated by these fibers or, as shown here, to changes in the input to the cortex from subcortical regions [8, 9]. As there is concomitantly a significant decrease in CMR_glc(ox)_ and hence the average firing rate in these areas [1], the question arises as to whether the decrease in CMR_glc(ox)_ is due to a loss in average firing rate contingent on the loss of correlations mediated by synapses formed by the associational fibers or to the input firing from subcortical regions? The cortical model presented here shows quantitatively how the decline in either the efficacy of the associational fiber synapses and/or the subcortical input changes the firing rates in cortical regions.

Given that the associational fibers connect widely separated cortical regions it might be anticipated that if a region receives a transient input from subcortical regions, such as the thalamus, this will be widely distributed in some form to other regions across the cortex, and this has been shown to be the case [10]. Our cortical model shows that different shaped transient BOLD signals occur in different modules following a transient box-car BOLD signal introduced into one module, and that these transients are the same as those observed across the cortex [10].

**Fig 1:**
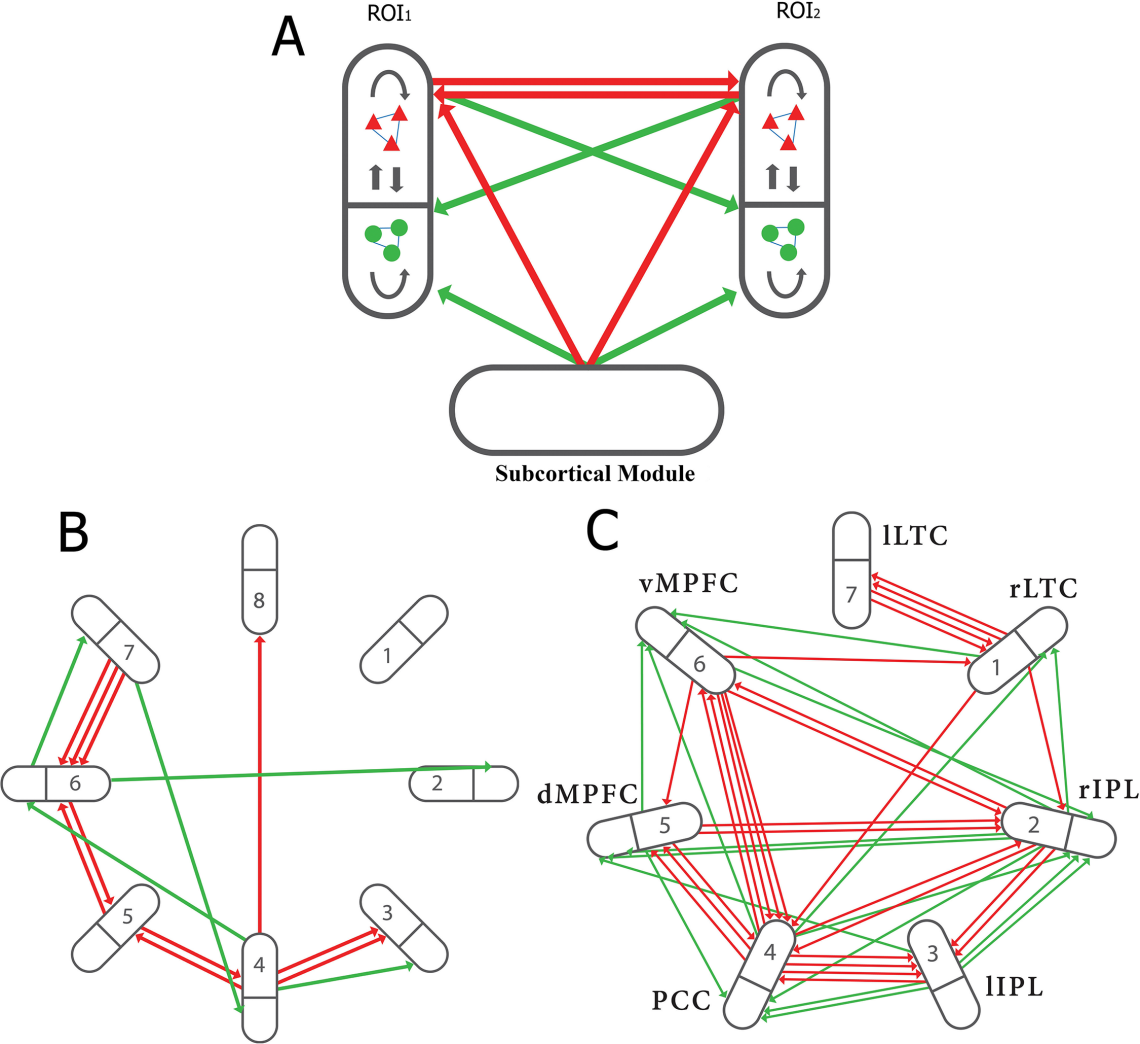
Shown are two modules (regions of interest, ROI_1_ and ROI_2_), represented by ellipses containing pools of excitatory neurons (red triangles) and inhibitory neurons (green circles), synaptically connected within and between pools (black arrows). The extrinsic (associational) axons between ROI_1_ and ROI_2_ originate and end on single neurons and are either excitatory to excitatory (red arrows) or excitatory to inhibitory (green arrows). The subcortical inputs are also excitatory; computationally, this input is represented by a Poisson train of firings, the strength and frequency of which can be varied. (B), extrinsic (intermodular, associational) connections in an 8 modular network (NI). The bold line within each module indicates separation of excitatory and inhibitory neurons within the module with all extrinsic fibers between the modules forming excitatory synapses, primarily on excitatory neurons within the modules but not exclusively so. (C), extrinsic (intermodular, associational) connections in a 7 modular network (NII). The connectivity due to the associational axons follows the criteria described in A above. NII can be taken to represent the Default Mode Network (DMN). In this case the modules may be identified as follows: 1, right lateral temporal cortex; 2, right inferior parietal lobule; 3, left inferior parietal lobule; 4, posterior cingulate cortex; 5, dorsal medial prefrontal cortex; 6, ventral medial prefrontal cortex; 7, left lateral temporal cortex. The number and diversity of the associational fibers were chosen so as to reflect the reported weight of such connections between the modules of the DMN (see [49], their Fig 8 and associated Table; also Fig 4A and 4C in [50]. (After Fig 1 in (15) with permission.)

Large scale networks of cortico-cortical anatomical connectivity have been used to analyze resting-state cortical activity [11–14] although phenomenological models of lower complexity are very useful in order to reveal important mechanisms responsible for the dynamics of cortical activity [11]. We have used such phenomenological models [15], described in the Methods and illustrated in Fig 1, in order to illuminate the mechanisms relating BOLD activity to the underlying firing rate in the different experiments outlined above.

## Materials and Methods

### Definitions

‘Associational fiber connections’ are extrinsic to the modules: axons making synaptic connections between one part of the cortex and another, considered as regions of interest (ROI).

‘Correlation of the BOLD signals’: correlation between the time course of BOLD signals in one ROI with that in another ROI.

‘Global signal’: the average BOLD signal across all ROI.

‘Structural connection’: a synapse.

‘Synaptic efficacy’: the probability of synaptic transmission.

### Cortical models

The network models we have used in this work have been described in detail in our recent publications [6, 15] and are only briefly described here. Modules in the networks, representing cortical areas, possess relatively large numbers of excitatory and inhibitory neurons (75% of the former and 25% of the latter, in agreement with observations of [16]) with recurrent synaptic connections. The time evolution of the network is computed by treating each neuron as an integrate-and-fire unit that sums inputs and generates an output firing when a threshold is reached (for details of the model, see (6). The ratio of excitatory to inhibitory connections to the excitatory neurons is 0.36. The inhibitory connections on the soma are very powerful in determining the initiation of firing and propagation from the soma, so we settled for an inhibitory to excitatory ratio of 0.36 on average. The strengths of these connections provided an excitatory to inhibitory ratio of strengths of 0.33 which may be compared with that reported in the literature of 0.5 [17] to 1.0 [18]. It should be noted that no attempt has been made in this work to reproduce the wide range of intermodular neuronal types and their connectivity that might lead to more appropriate patterns of firing than that observed in the present and previous modeling studies [6, 15]. The overall extent of intramodular connections, their absolute strengths and the total number of neurons per module were selected, given the above restrictions, so that the firing rates of isolated modules were neither continuous nor collapsed to zero.

The neurons in the network also receive an external background input of uncorrelated Poisson firing trains, like those that cortical neurons receive from subcortical regions such as the intralaminar nuclei of the thalamus, together with intermodular excitatory synaptic connections (compare with the network in [12]; see also [6]. The firing rate was transformed to BOLD signals using the model in [19] as used in [20] (see below). This model is currently used in all theoretical exercises involving determinations of the BOLD signal in relation to firings [12, 21], and there is good experimental evidence indicating a close correlation between these (see for example [22]).

The patterns of firing in the cortex are very irregular [23–25], with some showing bursts followed by relatively silent periods of about 0.5s [26] and this is observed in the present simulations. The patterns of firing are determined by the intrinsic properties of modular networks. Both the amplitude and frequency of the BOLD signals arising from the patterns of network firings, following application of the Friston equations [19], is similar to that identified experimentally (see Fig 2B in [27]) and in other cortical network simulations (see Fig 6 in [21]) in the literature.

**Fig 2:**
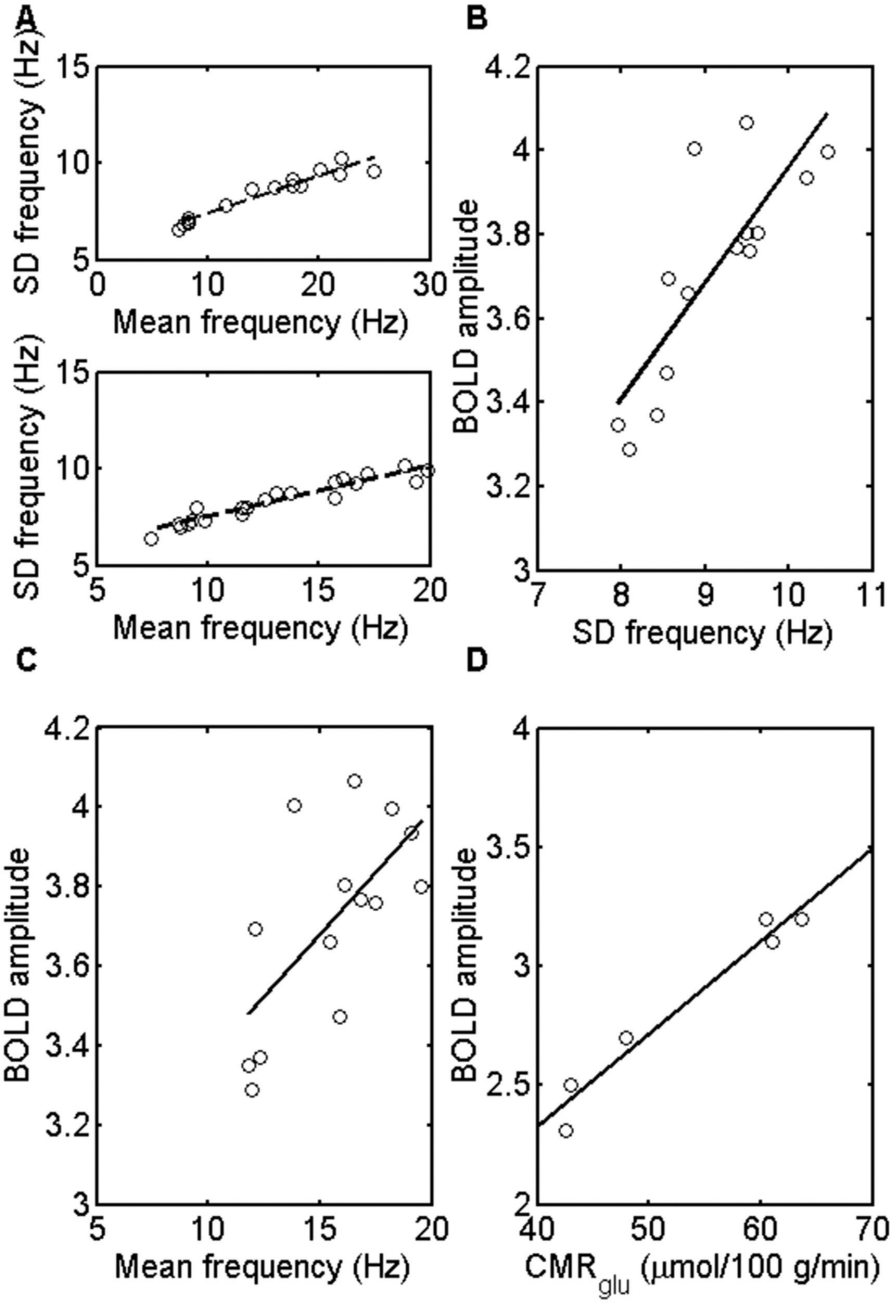
(A), correlation between the standard deviation (S.D.) of the firing rate and the mean firing rate in the modules of NI (upper panel) and NII (lower panel). These frequencies and their S.D.s were determined both for the networks given in the Fig 1B and 1C as well as for these with a variety of different extrinsic (intermodular) connection weights. (B), correlation between the amplitude of the RMS BOLD signals and the S.D. of the frequency of firing rates giving rise to the BOLD signals for NII in the resting state. The BOLD evaluations and the S.D.s of the frequencies were determined for NII as for the networks in (A); correlation coefficient 0.82. (C), correlation between the amplitude of the RMS BOLD signals and the mean frequency of firing rates giving rise to the BOLD signals for NII in the resting state. The BOLD evaluations and the firing rate frequencies were determined as for the networks in (A). The gradient of the regression line is 0.06 with correlation coefficient 0.66. (D), correlation between the experimental ‘resting state fMRI amplitude’ (BOLD amplitude) and the CMR_glc(ox)_ in the resting state in different brain regions of humans. The gradient of the regression line is 0.04, with correlation coefficient 0.98. Values are from Table 1 in [3].

### Single modules and systems of modules

The formalism for the network in a single isolated module, follows that of [6, 15], which in turn was based on [28] and [29]. The time evolution of the network is initiated by trains of independent but identically distributed Poisson inputs applied to each neuron. The complete system of modules consists of identical modules that are interconnected by a limited number of synaptic connections originating from single excitatory neurons and ending on either excitatory or inhibitory neurons, both connected neurons being randomly selected from the appropriate pool. For Network I with 8 modules the intermodal connections were generated randomly (Fig 1B), whereas for Network II with 7 modules the connections are based on data as given at the end of the legend to Fig 1 (Fig 1C). Simulation runs were generally of 300s duration, using a time step of 0.01ms and data sampling every 100ms. In the cases where extra stimulation was applied to a module, this was turned on at 25ms and then alternated 20s on and 20s off until the end of the run.

### BOLD signal

The BOLD signal is calculated from the neuronal activity using equations 3 and 4 of [19]; see also [21], equations 36–40. The equations developed by Friston and co-workers [19, 30] combine the Balloon-Windkessel model [31, 32] with a model of how synaptic activity causes changes in regional blood flow. The equations are, for a single neural region (module)

$$\dot{s}=z-\kappa s-\gamma (f-1)$$

$$\dot{f}=s$$

$$\tau \dot{\nu }=f-\nu^{1/\alpha }$$

$$\tau \dot{q}=f[1-(1-\rho {)}^{1/f}]/\rho -q\nu^{1/\alpha }/\nu$$

where $\dot{s}=ds/dt$, etc. The input to these equations is *z*, a measure of neuronal activity taken as proportional to the neuronal firing rate. *s* is a vasodilatory signal related to the blood inflow, *f*. ν is the blood volume, *q* the deoxyhemoglobin content and ρ the resting oxygen extraction fraction. The BOLD signal is given by

$$y=V_{0}[7\varrho (1-q)+2(1-q/\nu )+(2\varrho -0.2)(1-\nu )]$$

where *V*_0_ = 0.02 is the resting blood volume fraction. The other parameters have values κ = 0.65, γ = 0.41, τ = 0.98, α = 0.32, ρ = 0.34. A full discussion of these equations, their derivation and justification, can be found in Friston et al., [19, 30]. In our modeling the neuronal activity, *z*, that is the input to these equations, is obtained by multiplying the neuron firing rate (in Hz) by 0.01.

Sampling was carried out every 100ms over the 300s time series, with 50 samples averaged in general giving a bin of 5s and therefore 60 points on which to calculate the standard deviation (S.D.). This bin size was chosen as appropriate given the time course of a BOLD signal. In the case of the BOLD signal this S.D. gives the total BOLD fluctuations over the duration of simulation specified of the total signal, without prior regression of any global variation across the modules. Here the S.D. provides a summary of variability or power over all frequencies, particularly those in the low frequency range (i.e., above a period of five seconds).

## Results

### Cortical networks

Our networks consist of different numbers of modules, with each containing a hundred neurons, 75% excitatory and 25% inhibitory, having different extents of synaptic connections. Associational fiber connections occur between the modules with their own synaptic weights (for more details of this connectivity see Methods). Synaptic connections of neurons within any module may also originate from subcortical sources completely external to the network. In addition, another input to the network may make synaptic connections in relation to the synapses formed by the intermodular associational fiber connections whose efficacy they modify; these may originate for instance from subcortical regions such as the intralaminar thalamus. In order to simplify this network for computational purposes, the inputs external to the networks have been grouped and the synaptic efficacy of the intermodular associational synaptic connections changed according to requirements rather than arising from the intrinsic workings of the subcortical module in Fig 1A.

The projections within these modular networks are illustrated here by two networks with different levels of intermodular connectivity, designated network I (NI) and network II (NII), given in Fig 1B and 1C respectively, from which the external modules have been removed for clarity (for a more detailed description of these networks see [15]. NI consists of 8 modules (one in isolation for comparison with the rest), with each of these receiving between a single associational input from another module (modules 2 and 8), or inputs from 2 to 3 modules (module 6), with most (63%) of these inputs synapsing on excitatory neurons within modules, the rest on inhibitory neurons (Fig 1B). In contrast to NI, NII (Fig 1C) consists of 7 modules, all of which receive 2 to 8 associational fibers forming synapses onto excitatory neurons and from 0 to 2 associational fiber synapses onto inhibitory neurons. NI and NII have been chosen from a large number of modular networks we have investigated, as they best illustrate principles that emerge from this study of small networks.

### Changes in the extrinsic (intermodular, associational) synaptic connections lead to changes in BOLD signals in modules throughout the network that are proportional to the firing rate in the modules

BOLD signals that measure fluctuations in the resting-state firing rate in a region of cortex have to be reconciled with the observation that the BOLD is also proportional to the average firing rate or CMR_glc(ox)_ [3]. The S.D. of the firing rate in any module in NI and NII (Fig 1) is found to be highly correlated with the firing rate fluctuations (Fig 2A). This is true of both NI (correlation coefficient 0.99) and NII (correlation coefficient 0.98), independent of the extent of extrinsic (associational) synaptic connectivity between the modules in each of the networks. As the experimentally measured BOLD signal is taken to be proportional to the fluctuations in the firing rate, for example fast synchronous activity in the high frequency range [33] or particular patterns of bursting or transitions between up and down states, it was necessary to confirm that this is the case in the networks under consideration. Fig 2B shows that it is approximately the case, with a correlation coefficient of 0.82 relating BOLD amplitude, computed over 60 five second windows, to the S.D. of the firing rate for NII modules. However, as the S.D. is linearly related to the firing rate in these networks (Fig 2A) it follows from Fig 2B that the BOLD amplitude should be linearly related to the firing rate, and this is shown to be the case (Fig 2C) with correlation coefficient 0.66. This implies that the BOLD signal as normally determined provides a relative measure not only of the firing rate fluctuations but also of the average firing rate, as has been shown experimentally to be the case (Fig 2D). The gradient of the regression lines relating experimentally determined BOLD amplitude to CMR_glc(ox)_ is 0.04 (Fig 2D), compared with the theoretical gradient of 0.06 (Fig 2C). These relations are likely due to the nature of the on-going bursts of firing activity in the modules (examples of which are shown in the Supplementary Information in [15]. In this case, the observed relations between the mean and S.D. of firing activity arises as a consequence of this pattern of activity. The S.D. provides a summary of variability or power over all frequencies, particularly those in the low frequency range (i.e., above a period of five seconds).

**Fig 3:**
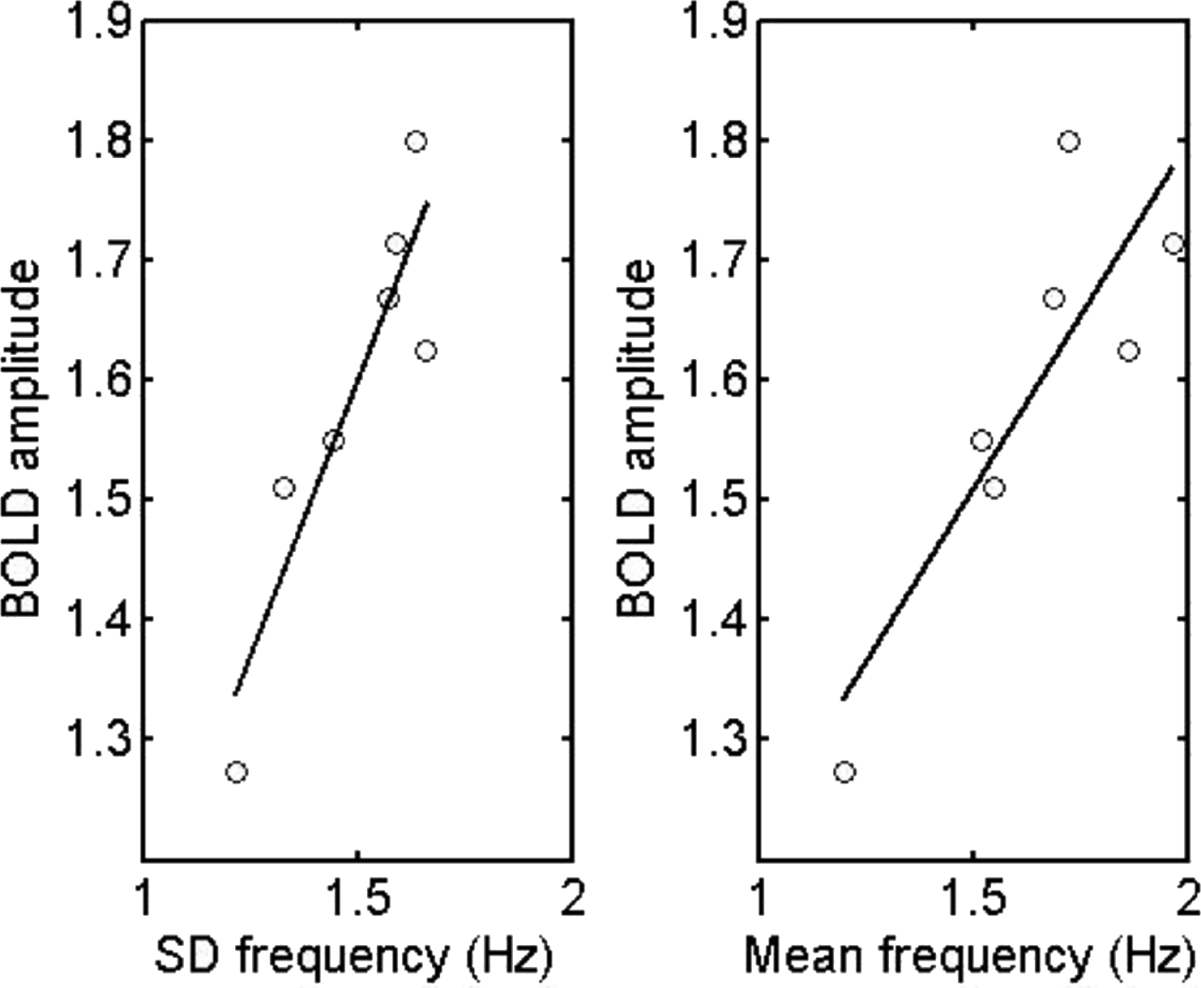
The BOLD amplitude is linearly related to both the mean firing rates and their S.D. down to frequencies in the range of those observed in the cortex of primates. Dependence of the BOLD amplitude on the S.D. of the firing rate (A) and of the mean firing rate (B) when this was reduced by decreasing the subcortical input to the modules until the mean frequency was less than 1.25Hz while keeping the extrinsic (intermodular) connection weights constant. The linear relation between the BOLD amplitude on the one hand and both the mean firing rate (B) and the S.D. of this (A) on the other is maintained as the frequency is reduced.

The dependence of the BOLD amplitude on firing rate can be scaled down to much lower mean frequencies, as it is likely, although uncertain, that the firing rate in humans are substantially less than 10Hz. This scaling down of the frequency to much lower firing rates is accompanied by a scaling down of the S.D. of the frequencies (Fig 3A and 3B). The results of Fig 3 were obtained by changing the firing input to the network, taken to originate from subcortical areas, and not by varying the extrinsic (associational) synaptic connectivity as for the results in Fig 2. Thus the linear relation between the BOLD signal on the one hand and both the S.D. and firing rate on the other is not dependent on how these are varied by modifying the network, at least not for the range of values given in the cases considered.

### Loss of BOLD correlations between modules, mediated by the extrinsic (intermodular, associational) connections, is achievable with a decrease of firing rate within modules

Correlated BOLD activity between regions of cortex can be modeled by networks such as NI and NII (Fig 1B and 1C). The network NII can be used to model the correlations observed between different regions of interest in the cortex, both in awake and in Non Rapid Eye Movement (NREM) sleep as well as following anesthesia [15]. The loss of correlated activity between certain regions of the cortex in sleep and anesthesia is accompanied by CMR_glc(ox)_ decreases of about 40% in these regions [1], implying a 40% decrease in firing rate in these regions, given the strong linear relation between firing rate and CMR_glc(ox)_ [1]. The question arises as to whether there is a causal relation between the loss of BOLD correlations between regions and the decrease in firing rate in these regions?

**Fig 4:**
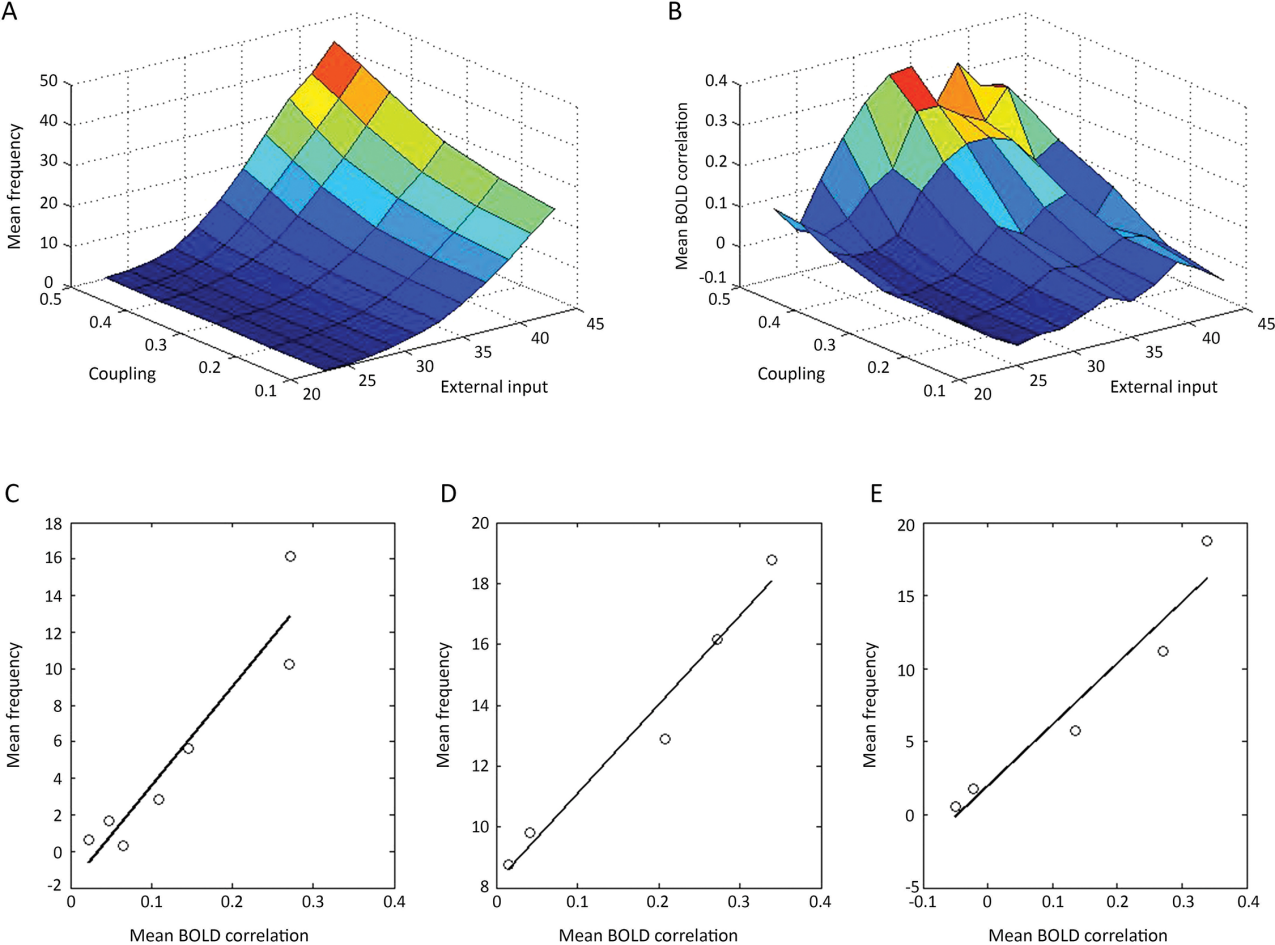
The relation between the mean firing rate in modules of NII (Fig 1C) and the mean BOLD correlations between the modules as either the coupling strength between modules or their subcortical input frequency are changed or both are changed at the same time. The changes in average firing rate (A), and the average BOLD correlations between modules (B), are shown for changes in the subcortical input frequency and coupling strength. The changes in the average firing rate versus changes in BOLD correlations when these are altered by changing only (C) the subcortical input frequency (25 Hz to 35 Hz) at constant connection strength (0.45) or (D) the connection strength (0.2 to 0.5) at a constant input frequency (35 Hz) are given by the graphs. The gradient in C is 54 Hz and that in D is 29 Hz (linear regression lines drawn, with in (C) y = 54x – 1.9 and in (D) y = 29x + 8). In E the changes in the average firing rate versus changes in BOLD correlations are given when these are altered by changing both the subcortical input frequency and the coupling strength simultaneously, a linear relation between these being assumed. The gradient of the line in E is 41 Hz (y = 41x + 1).

The mean firing rate in the modules following changes in the average coupling strength between the modules or changes in the frequency from the subcortical input are given in Fig 4A. The mean BOLD correlation changes, for changes in coupling or external input frequency, are given in Fig 4B. These results allow graphs to be drawn for the relation between mean frequency and mean BOLD correlations for the situation where these parameters change as a consequence of changes in the subcortical input frequency (Fig 4C) or changes in the coupling strength (Fig 4D). The linear gradient of the former is much steeper than that of the latter (54Hz compared with 29Hz), so that much greater changes in the mean firing rate are associated with changes in the mean BOLD correlation following a change in the subcortical input, than with a change in the coupling strength. In the model a 35% decrease in the firing rate, of the kind observed in some parts of the cortex during NREM sleep, can be obtained together with changes in mean BOLD correlations comparable to those observed [9], with a change in the subcortical input. For example, a 35% drop in the firing rate in the modules accompanies a 28% drop in the BOLD correlations following a drop in the subcortical frequency. This may be compared with the observed changes in the average correlations in the sensorimotor network from the awake state to deep NREM sleep of 25% (Fig 1A in [34]). On the other hand, changes in the coupling strength required to produce an average 35% reduction in firing rate in the modules are associated with very large percentage changes in mean BOLD correlation, of the order of 70%, that are not observed ([9]; [34]). While this argues for changes in the subcortical input frequency input providing the most likely basis for the changes in firing rate and BOLD correlations observed under a variety of experimental conditions, such as during NREM sleep, it certainly does not preclude a judicious mixture of both changes in extrinsic (associational) fiber coupling and subcortical input frequency. We have therefore looked at cases in which both the subcortical input and the coupling strength are changed simultaneously. Fig 4E gives the relation between the firing rate and the BOLD correlations for the case when the subcortical input frequency and coupling strength are linearly related, so that the coupling goes from strengths of 0.2 to 0.5 as the subcortical input frequency goes from 25Hz to 35 Hz. In this case, as anticipated, the gradient relating frequency to BOLD correlations is intermediate, 41Hz, to that of the other two gradients (compare Fig 4E with Fig 4C and 4D).

### Extrinsic (intermodular, associational) connections mediate transient changes in BOLD signals throughout the network following a transient change introduced into a single module

**Fig 5:**
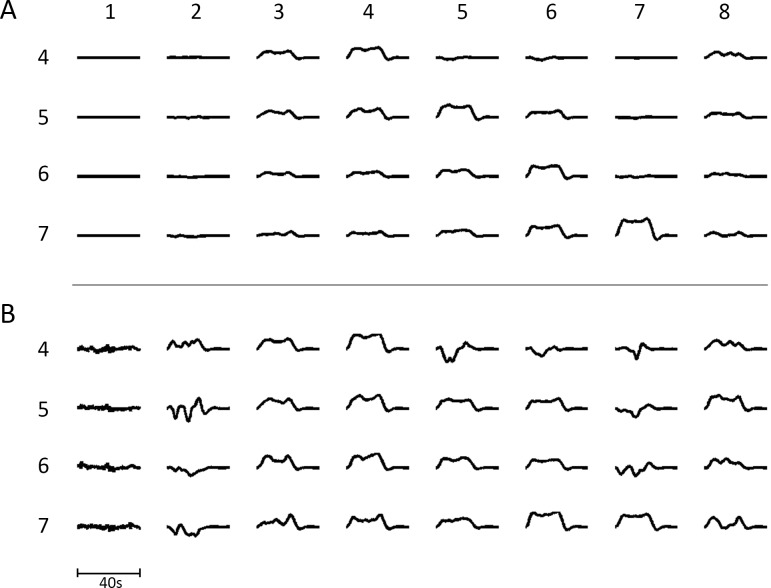
(A) The transient BOLD signal in each module in NI (columns numbered 1 to 8) following successive 20s increases in the subcortical input to modules 4 to 7 (rows numbered 4 to 7), causing increased firing in these modules. The input was simulated by increasing the subcortical input for 20s periods. (B) gives the same results as in (A) except that individual transients are amplified sufficiently to observe them in each of the 8 modules. This figure should be compared with that of Fig 4C in [10] in which similar experimental transients were observed over the cortex in a simple psychological test.

We determined if the modules responded to transient increases in firing input from an external source, representing subcortical inputs. To this end, each of the modules in NI was given in turn an extra transient average input for 20s and the BOLD response in each of the modules throughout the network measured (see Fig 5A). Following transient increases in modules 4, 5, 6 and 7, respectively, BOLD transients could be observed, mostly of the boxcar shape, in modules 4, 5 and 6. However, responses in all the modules throughout the network could be identified with sufficient amplification (Fig 5B). Many of these modules gave BOLD responses that had very different shapes to the boxcar variety: for example those in modules 2 and 7, which receive extrinsic (associational) synaptic connections that primarily synapse on inhibitory neurons within the modules. The distribution of the BOLD transient shapes and sizes is very similar to that observed experimentally where responses with the shapes given in Fig 5B are identified over widely spaced regions of the cortex after suitable averaging to improve the signal-to-noise ratio [10].

## Discussion

### BOLD signals are proportional to both the mean and S.D. of the firing rate in the resting state

The subcortical input to each neuron in the network of modules gives rise to bursts of firings in the modules (see Supplementary Information in [15]), that have similar characteristics to those of ‘up-states’ recorded in neocortex [35, 36]. Indeed it is known that a spontaneous up-state in one module can initiate bursts in other modules [37, 38]. This average frequency increases with an increase in the frequency of the subcortical input for each module, whether or not it is embedded in a network of modules [15]. As a consequence of this pattern of activity the standard deviation of the firing rate is proportional to the average firing rate (see Fig 2A). This arises as follows: let the firing rate within a burst be *b* Hz and let ℓ s be the length of the burst. To a good approximation, we can take *b* and ℓ to be constants. If *p* is the probability of a burst occurring in any given interval, then the number of bursts *X* occurring in *n* intervals each of length ℓ is binomially distributed with mean *np* and standard deviation $\sqrt{np(1-p)}$. It follows that the overall firing rate *Xb/n* is also binomially distributed with mean µ = *pb* Hz and standard deviation $\sigma =\mu \sqrt{\left(1-p)/(pn\right)}$ Hz. Note that as well as showing that the standard deviation σ is proportional to the firing rate µ, this also shows that σ decreases as the inverse of the square root of the length of the time window *n*ℓ s used in analyzing the simulation data. In the present work sixty windows of 5 s were used in the calculations.

### Loss of average resting-state firing rate over modules during sleep and anesthesia associated with loss of BOLD

During NREM sleep there is a fall in BOLD correlations between many areas of cortex although there are some increases in others [39, 40]. The greatest decline, of over 70%, occurs between the medial prefrontal cortex and the posterior cingulate cortex and also between the medial prefrontal cortex and the right inferior parietal lobule (see Table 2 in [41] and also [42]). This reflects the fact that NREM sleep is accompanied by fewer long-range effective associational connections with increased ‘cliqueness’ of local connections (that is there is a clustering of local ‘sub-modules’) [39, 40, 43]. Given the loss of these BOLD correlations there should be a significant decrease in firing rate in individual modules according to our model, reflected by a decrease in CMR_glc(ox)_ in the modules, and this is observed [1]. This global drop in firing rate, accompanied by a decrease in BOLD correlations, can be most easily accommodated in the present model by a decrease in the firing rate of subcortical inputs to the modules rather than by changes in the connective strength between them, although both might be involved with the former having a much greater impact on the changes in firing rate in the modules and the associated changes in BOLD correlations. Furthermore the possibility of an increase in the spontaneous down-states in the individual modules, caused by local changes in the efficacies of the intrinsic intramodular synapses during NREM sleep or anesthesia, cannot be discounted.

It is of considerable interest that during quiet wakefulness transcranial magnetic stimulation (TMS) of the premotor area leads to an initial electrical response there, followed by a sequence of waves that propagate to other areas of the cortex with which the premotor area has long associational connections. This is in contrast to NREM sleep when the electrical response at the premotor site of stimulation is strong but does not propagate to other sites at all [41, 42]. These observations are consistent with the idea that the principal decrease in firing rate and BOLD correlations under these conditions is due to a decrease in the efficacy of associational synaptic connections that block the propagation of the cortical waves [44].

Propofol anesthesia also reduces BOLD correlations [45, 46]. For example, the BOLD correlations of the medial prefrontal cortex are reduced from 0.6 to 0.1 and those of the posterior cingulate cortex from 0.6 to 0.3 under propofol. This is in contrast to the auditory and visual resting-state networks where the BOLD correlations are minimally altered [8, 45, 46]. These decreases in BOLD correlations are accompanied by decreases in CMR_glc(ox)_ of about 50% to 60% in different areas of cortex [47], as expected from the relation between decreases in the firing rate in different modules and their loss of BOLD correlations (see Fig 4A and 4B).

Another interesting aspect of the effects of changing the resting-state activity in sensory areas with an anesthetic is that a transient input from the thalamus gives rise to a more spatially confined BOLD change in sensory cortex, although the BOLD signal is much enhanced in amplitude [48]. This is to be expected from the model if an effect of the anesthetic is to decrease the associational fiber connectivity, for in this case some cortical areas (or modules) are isolated from others giving then a spatially confined BOLD signal. Furthermore, since the area (or module), as a consequence of being isolated, has a low level of resting-state firing activity then it will give rise to a much larger BOLD signal in response to a given transient subcortical (thalamic) signal (Fig 4 in [15]).
